# Exploring the potential of virtual reality technology to investigate the health and well being benefits of group singing

**DOI:** 10.1080/14794713.2018.1558807

**Published:** 2018-12-27

**Authors:** H. Daffern, D. A. Camlin, H. Egermann, A. J. Gully, G. Kearney, C. Neale, J. Rees-Jones

**Affiliations:** a Audio Lab, Department of Electronic Engineering, University of York, York, UK; b Royal College of Music, London, UK; c York Music Psychology Group, University of York, York, UK; d Stockholm Environment Institute, Environment Department, University of York, York, UK

**Keywords:** Virtual reality, singing, performance, well being, virtual acoustics, choir singing

## Abstract

There is a growing body of academic research aiming to quantify and understand the associated health and well being benefits of group singing. The social interaction is known to strongly contribute to perceived improvements to mental and physical health but there are also indications that singing together elicits better well being outcomes than other community activities. This paper introduces the Vocal Interaction in an Immersive Virtual Acoustic (VIIVA) system, which allows the user to take part in a group singing activity in 360 degree virtual reality, hearing themselves in the recorded venue alongside the other singers. The VIIVA is intended to make group singing accessible to those unable to attend real community choirs but also as a tool for experimental research into the health and well being benefits of group singing. This paper describes the VIIVA system and presents a number of methodologies and applications which are discussed in relation to three ongoing research projects. Preliminary work indicates that the VIIVA system provides a promising tool with which to study the health and well being benefits of group singing, and in particular to control for the social interactions inherent in real group singing activities.

## 1. Introduction

The popularity of group singing is increasing within communities, and likewise Virtual Reality (VR) technologies are becoming more readily available to the home user. This paper outlines the ongoing research of an interdisciplinary team utilising VR technologies to widen engagement and increase understanding of group singing performance, using an interactive virtual reality system for ensemble singing, known as the Vocal Interaction in an Immersive Virtual Acoustic (VIIVA) system.

### 1.1. Group music performance using digital technology: background

Various pathways have been explored to enable remote group music making, some of which focus on exploiting available digital media to enable real time collaborative performance, whilst others exploit the recording potential of digital technologies for artistic endeavours.

There is a clear appetite amongst musicians for commercial products such as the bespoke software, *ejamming* (http://www.ejamming.com), which provides audio-based software for musicians to perform together in real-time from remote locations (Blythe and Sewell 2011). An overview of live remote performance using internet networks was provided in Carôt, Rebelo, and Renaud (2007), which includes the issues of latency (the delay between the transmission and receiving of data), one of the most pertinent limitations of current technology for remote collaborative performance, and the approaches of different systems to either combat or allow for latency with a system.

Other examples whereby digital technologies have been implemented to enable remote musical learning environments include masterclasses (Enloe, Bathurst, and Redmond 2013), and one-to-one instrumental lessons using conference technologies (e.g. Kruse et al. 2013). The challenges facing the collaboration of remote musicians in real time have been scrutinized through the development of Distributed Immersive Performance (DIP), which utilises high resolution video and audio with frequency and spatial content preserved in playback, to allow musicians to perform together in real-time via a flat screen and loudspeakers (Sawchuk et al. 2003). The system was first designed for a streamed symphony orchestra performance, then developed for a masterclass situation, and also for a two-way interactive collaboration (Chew et al. 2004). Issues such as latency were approached practically, for example, by introducing an equivalent digital delay in piano duet performance and assessing performers' experiences (Chew et al. 2006). This system has not been tested with singing as far as the authors are aware.

Specifically for multiple voices and remote rehearsal, choirs have reported utilising commonplace internet communication technologies for rehearsals, however with notable limitations and challenges when working ‘live’. Near North Voices found the set-up time for hardware and latency of Skype and Google Hangout to be problematic, preventing the conductor from rehearsing the choir remotely and instead using this technology to facilitate a coaching role, removing the requirement for real-time interaction during performance (Adler 2014). Even with these limitations the choir found beneficial ways for choristers and the conductor to use the technology to connect remotely to rehearsals, commenting that ‘Choristers can even sing along with the choir, so long as they turn their microphone off to prevent temporal interruption for the choir, and they are able to ask questions during the rehearsal’ (Adler 2014). Ultimately, the director found it useful to record the rehearsals to allow choristers to ‘catch up’ on missed rehearsals when ‘live’ remote access wasn't possible.

An international project also centred around singing rather than instrumental performance is Eric Whitacre's Virtual Choir, which has proven to be hugely popular. The Virtual Choir brings together singers from across the globe through video recordings which can be uploaded and then edited into the ‘performance’. Since its first inception in 2009, Whitacre has worked on several Virtual Choir projects engaging thousands of singers from hundreds of countries (Whitacre 2018). Whilst the first two projects were recording-based, in 2013 the Live Virtual Choir was realised with 30 singers from 30 different countries over Skype, with the piece ‘Cloud Burst’ being adapted to specifically account for the latency (Whitacre 2018).

These projects illustrate the growing interest amongst musical communities to embrace digital technologies in their practice. The rapid advances being made in the areas of digital media and communication technologies in parallel with a willingness to engage with these technologies provides a great opportunity for them to be exploited further.

### 1.2. Singing together for health and wellbeing: background

The strong impetus to enable group performance across society through technologies, is echoed in current attitudes towards traditional ‘real’ group music making, especially with a renewed enthusiasm for community singing projects. The increasing popularity of community choirs is likely to be linked to the associated health and well being benefits of singing, especially group singing, which are being extolled informally across popular media outlets (Eno 2008; Lacey 2013; Morelle 2013; Burkeman 2015) and are of increasing interest in academic and health communities.

Initial studies indicate notable psychological benefits of group singing, including significant clinical improvements on mental wellbeing for people suffering ongoing mental health issues (Clift and Morrison 2011) and self-endorsed improvement effects in a community choir (Clift and Hancox 2001; Clift et al. 2013). Other studies using qualitative research methods have indicated a positive correlation between well being and engaging in choral singing situations, including in community projects and in aging populations (Clift and Hancox 2001; Hillman 2002). There are few quantitative studies into the physical benefits of choir singing, however researchers are starting to investigate physiological responses which are connected to stress, such as cortisol and immunoglobulin levels (Kreutz et al. 2004). Significant reductions in negative affect and increases in positive affect, alongside significant increases in cytokines and reductions in cortisol, beta-endorphin and oxytocin levels have also been reported in choir singing (Fancourt et al. 2016).

Technologies are now emerging which allow data to be collected during singing, allowing the possible interpersonal effects of choir singing to be assessed, with preliminary findings suggesting that breathing rate and heart rate synchronise during group singing tasks (Vickhoff et al. 2013). Whilst progress is being made, empirical research in this area is still in its infancy, with an acknowledged need for robust interdisciplinary studies (Stacy, Brittain, and Kerr 2002; Gick 2011).

Although empirical evidence in the area is still lacking as described above, group singing has become popular as an informal complementary treatment for various conditions, for example with the emergence of Parkinson's Choirs. Speech therapy, in particular to increase the volume of the spoken voice, is common for Parkinson's patients, attributed in part to a change in auditory feedback (Ho et al. 1999) combined with a symptomatic bowing of the vocal folds (Blumin, Pcolinsky, and Atkins 2004). Increased jitter and shimmer are also common in Parkinson's patients' spoken voices (Jiménez-Jiménez et al. 1997). Preliminary studies (Di Benedetto et al. 2009; Evans et al. 2012; Shih et al. 2012) have found benefits of choral singing as a treatment for Parkinson's disease. Singing has also been implemented as an additional form of speech therapy, with an integrated voice and choral singing treatment showing promising results, including improvements in phonation time and voice fatigue (Di Benedetto et al. 2009).

Research into choral singing also indicates that when singing together, individual voices may have a direct impact on each other in terms of the voice production itself, which could have implications on vocal health benefits of group singing. The ways in which voices adapt to each other (in terms of vocal fold function and acoustics) is not really understood beyond pitch matching (e.g. Ternström and Sundberg 1988; Ternström 2003), however, perceptual factors such as the Lombard effect (the phenomenon that individuals will increase their volume with their surroundings) are known to be relevant in choirs (Tonkinson 1994). Such perceptual theories could in part explain the improvement observed by Parkinson's patients when participating in group singing activities: singing in a choir provides the same auditory masking that is often simulated in speech therapy to encourage patients to increase voice intensity (Adams and Lang 1992).

Whilst group singing is known to be beneficial, many populations cannot engage with the activity, due to either physical or practical limitations of the individual. Indications of the research discussed above suggest that the very groups isolated from group singing activities are those who might benefit most. At the same time, the growing appetite for remote engagement with music performance is evident in the examples described in Section 1.1 across both live and recorded technologies. There is a great opportunity with the rapid advances in VR technology to create immersive group performance systems to improve the health and wellbeing outcomes for certain user groups.

Exploring the potential application of the VIIVA system for health and wellbeing is at the forefront of the current research projects which implement the system. The main avenues of research to this end are three-fold: firstly, to develop the system to improve access to group singing as a remote activity, eventually through home technologies; secondly, to understand how users engage with singing in this environment compared to a ‘real’ situation; and thirdly, to use the system as a tool for controlling experimental environments to improve understanding of the health and wellbeing benefits of group singing.

In the following sections of this paper the implementation of the VIIVA system, developed to explore the potential of VR to engage users in a virtual interactive group singing activity, is described. This is followed by a description of a number of measurement tools that might provide insight into the potential health and well being benefits of group singing, with their feasibility within experimental designs and applications then discussed in relation to three ongoing research projects.

## 2. Vocal Interaction in an Immersive Virtual Acoustic (VIIVA) - an interactive VR system

The VIIVA system was developed to capitalise on the growing interest in VR technologies within the performance and research communities, in order to improve access to group singing situations but also as a tool for data collection and research into the effect of singing on health and wellbeing. A key aim of the VIIVA system is to provide an innovative tool for controlling variables in experimental designs based around social activity, which is an important step in being able to understand the benefits of singing as a group experience.

The VIIVA system allows the user to take part in a pre-recorded group singing experience, whereby they take on the role of a missing singer within the group. In addition to seeing and hearing the recorded performance in 360 degrees from the perspective of a performer, the system provides interactive audio, in that as the user sings they also hear themselves in their position within the original recording venue.

The prototype system used a 360° camera rig and spherical microphone array to ‘replace’ a singer in the vocal quartet. This required the piece to be recorded four times in the original environment (a church), with one part (soprano, alto, tenor, bass) missing each time, and the remaining three singers interacting with the recording equipment as if it were the missing singer. Room impulse Response (RIRs) measurements were taken from each position in four rotations to allow position specific spatial auralisation of the venue. The data capture and technical implementation of the prototype system are discussed in full in Kearney et al. (2016), and are explored further in Section 4.1.

The system is immersive in that the user/performer appears within the relevant group singing setting, observed visually and aurally through the headset and headphones/speakers in 360 degrees. Whilst standing in place as a member of the choir the user can move their head and observe the recorded activity around them, retaining the spatial information in the experience. Established categories of immersion for VR (e.g. Bystrom, Barfield, and Hendrix 1999) are achieved in this system, such as exclusion to the real world (as the user wears a headset), and being extensive and surrounding, in that visual and auditory rendering is delivered in 360 degrees; however, a ‘virtual body’ is not included in the system to date (if the user looks down they will not see their body), a limitation which the authors acknowledge reduces the validity of the term immersive in the context of VR (Usoh and Slater 1995). This will in turn have an impact on the experienced ‘presence’ of the system, defined by Slater as ‘the qualia of having a sensation of being in a real place’ (Slater 2009). Concepts of immersion reported in gaming literature suggest that a sense of being in the ‘task environment’ with a loss of awareness of time and the real world contribute to immersion with emotional involvement in the activity also an important factor (Jennett et al. 2008). The spatial audio delivery of the VIIVA system is therefore particularly important, not only because of the musical context of the experience but because sound delivery has been shown to have a significant effect on a user's sense of presence (Sanders and Scorgie 2002).

It is important to note that the user cannot move around the recorded space: their position is static apart from the head rotation. In light of the theory that place illusion ‘occurs to the extent to which participants probe the boundaries of the system’ (Slater 2009, 3552), the VIIVA experiences have been devised to maximise the place illusion within the limitations of the current system. This includes, promoting physical stillness through the singing activities of the recordings, i.e. no large movements / actions are performed by the other performers or conductors during songs or warm-ups, and songs are always performed without a score, relying on singing from memory or taking instruction from the conductor where appropriate. Users were ‘encouraged’ to maintain eye contact with other performers or watch the conductor in order to reduce the desire to look down (and not see their body). This is general practice in group singing activities and was promoted by having the performers engage with the video camera during the recording as though it were part of the performance, and through placement of the recording equipment in the setting (embedded within parts where possible).

The VIIVA is considered interactive due to the audio interaction, however, the user cannot interact with the other performers in the current system as it is pre-recorded. Therefore, the other singers in the situation will not react to the user. These factors will undoubtedly reduce the ‘plausibility illusion’ as defined by Slater, which refers to the user accepting that the VR situation is actually happening, which will likely impact the perceived ‘realism’ and sense of immersions of the system for the user (Slater 2009).

### 2.1. Responses to the VIIVA system

The singers involved in the original church recording returned to test the prototype VR system, performing their own part within the virtual environment wearing an Oculus Rift headset and with audio played back over a horizontal eight-speaker array. The ‘virtual’ singer's voice was captured using a head-mounted microphone and auralised using the impulse response captured at each singer's position during the original recording procedure. Participants were asked for informal feedback in addition to objective measures of engagement, emotion and vocal fold behaviour as detailed in Section 5.2.

All four singers reported enjoyment when using the system and found it to be highly realistic, in that it was like the real experience, with comments such as ‘I feel like I'm really there’. The researchers were concerned about the lack of peripheral vision provided by the Oculus headset, particularly in light of research which suggests that fields of view of visual displays are a particularly important feature in immersive systems (Cummings and Bailenson 2016), but participants did not comment on this until prompted, whereupon they reported moving their heads more than usual to compensate.

The system was updated to use an HTC Vive headset, with a slightly wider field of view than the Oculus Rift (110 degrees rather than 100 degrees) and both experienced and inexperienced singers who had not performed in the original space or with the original singers were invited to try the experience. All users were invited to try out the experience first as a solo singer, standing alone in the church venue in a performance position to assess their response to the interactive spatial audio. Unless they knew the four-part piece from memory (in which case they could fill in their missing part), for the group singing experience, users replaced the singer which represented most closely their voice type (soprano, alto, tenor or bass) and they took part in a unison quartet performance of *Amazing Grace*.

Similar to the feedback of the original singers, these users reported that they engaged well with the system, commenting on the experience feeling ‘real’. However, there were also comments about feeling inhibited singing the space: several users, one who regularly performs in amateur choirs and classed themselves as ‘experienced’, reported that they felt intimidated by how good the other singers were. This could indicate a good degree of presence experienced by these users, and when discussed, comments such as ‘I know they can't really hear me but I still feel shy’ were common. Considering a key application of this system is to encourage group performance across communities, this needs careful consideration in the design of VIIVA experiences. Whilst participants were asked to discuss the quality of the VR experience in terms of how ‘real’ it felt there was no formal methodology employed to measure immersion or presence, although the authors acknowledge that this is an important area for further study.

### 2.2. Current system, limitations and future development

**Figure 1.**
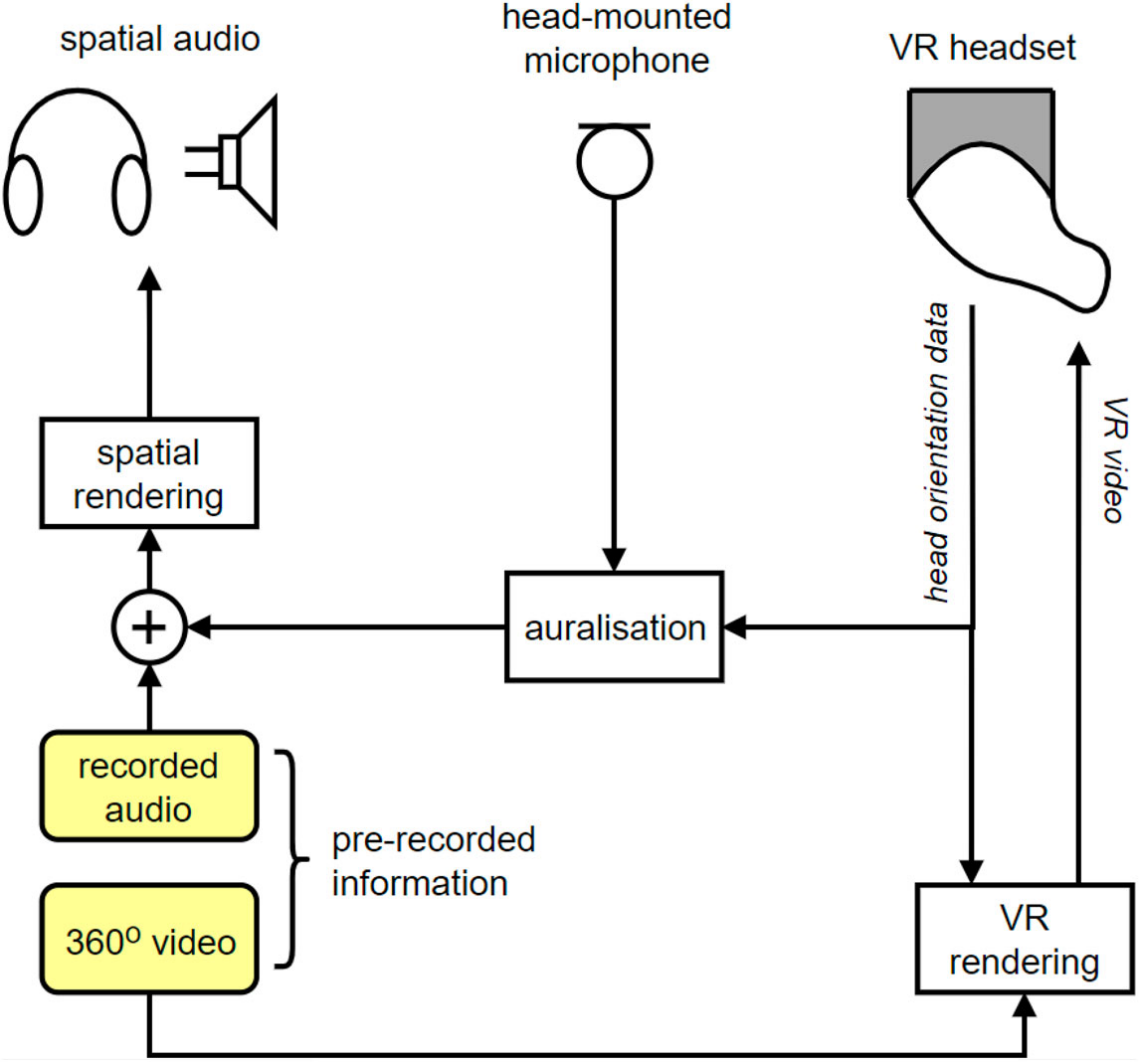
General outline of the VIIVA system.

**Figure 2.**
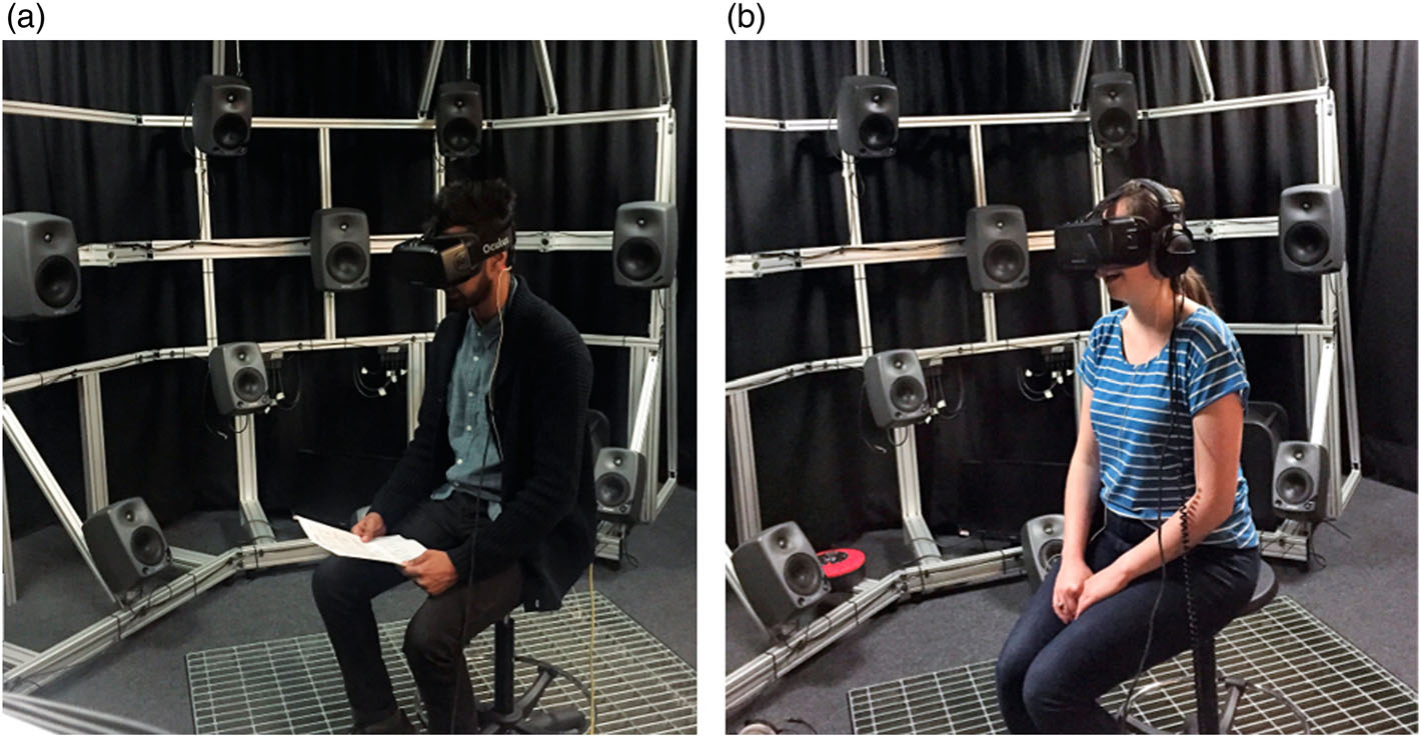
(a) Loudspeaker based auralisation with singer in-situ wearing VR headset and head-mounted microphone; (b) same scenario with headphone listening (Civit 2017).

The general outline of the current VIIVA system is illustrated in Figure 1. The current version of the fully interactive VIIVA system at the time of writing continues to make use of the HTC Vive headset, but has been enhanced for greater levels of auditory immersion through reproduction over a 50-channel spherical loudspeaker array, using higher-order Ambisonics rendering (Lecomte et al. 2015), as illustrated in Figure 2(a). Alternatively, spatial audio may be reproduced over headphones, using binaural sound reproduction, as illustrated in Figure 2(b), with the latter system tested and validated against the former (Civit 2017). The headphone-based system additionally compensates for tonal colouration due to the microphone, headphones, and influence of the headphones on the singer's perception of their own voice; this aspect is particularly important as headphones not only affect the perceived tonal balance of the voice, but may also impact on haptic feedback via bone conduction. This work has shown that equalisation filters are essential in the headphone-based system to improve naturalness and more closely match the loudspeaker-based reproduction.

Utilising the modifications which have been made to the system, and particularly implementing the experience through headphones, a version of the VIIVA has now been implemented using Samsung Gear (see Section 5.4) and future plans include its development to allow easy installation in the home across different hardware platforms.

Theories of presence in VR, such as those summarised and integrated into a model of immersion, presence and performance by Bystrom, Barfield, and Hendrix (1999) highlight some of the limitations of the current system. In particular they illustrate the need for avatars to be employed within the experience to include a ‘virtual body’ for the user and the sense of place experienced by allowing the user to move around the space.

The long term goal for the system is to enable a fully interactive VR system, through the use of avatars, able to reproduce the acoustic, reflexive and social dimensions of group singing. There is much work to be done before this ambition can be realised, especially the issue of latency across internet connections which were discussed in relation to existing real time systems in Section 1.1.

## 3. Recording setups

Using VR technology to enable the practice of group singing, both as an activity in itself and as a tool to engage in experimental research requires careful design of VIIVA experiences to ensure delivery appropriate to the purpose of the project. For use by non-singers to encourage engagement with the activity (i.e. improving confidence in group singing), for example, a larger choir performing simple repertoire would be required. This section describes the necessary considerations for the creation of the VIIVA experiences.

### 3.1. VIIVA stimuli: recording the VIIVA experiences

The objectives of a given project will determine the specific needs of the VIIVA experience and how that experience should be obtained. As the current system is a rendering of 360 degree recordings, not a fully virtual system with avatars, a bespoke recording needs to be made for every required participant position.

In order to make the experience as immersive as possible, whereby the user feels as though they are part of the group in the recording, the positioning of the camera is particularly important. The user can't move around the VR experience, so their static position needs to give them the impression of being within the group rather than observing from outside, therefore the camera needs to be placed in the space a singer would normally occupy.

Height positions for the system have been approximated based on average adult height to account for multiple users. Ideally the microphone would be positioned at ear height, however the camera rig also needs to be placed at eye height. Placing the microphone and camera side by side would result in the camera being seen as the user turns their head in the VIIVA experience. The microphone was therefore placed just underneath the camera (the user looking down in the VIIVA experience sees a tripod rather than their body).

During recordings singers were always instructed to interact with the recording equipment (the microphone and video camera) as if it were the missing singer. This did not appear to pose a problem in a quartet of semi-professional singers, and in the larger groups that have been recorded it was not an issue as there was a conductor as the visual focus for the choirs. Interaction between singers is a key element of group singing and is likely to affect the quality of the VR experience, as realistic interactions within a VR environment are connected to the level of presence experienced by the user (e.g. Bystrom, Barfield, and Hendrix 1999).

In addition to the live recording, in order for the audio to be interactive and spatially realistic, RIRs need to be obtained from each VIIVA perspective / microphone position. This has implications on the resource needed to undertake research using this system, as this is time consuming and requires heavy equipment.

## 4. Measurement tools

Using wearable technology in the form of sensors, it is now possible to measure various physical parameters during singing, rather than relying on pre-and post activity data collection. These include a number of quantifiable measures associated with engagement, and health and well being, some of which have been employed in previous work on group singing (e.g. Vickhoff et al. 2013). Ideally it is now possible therefore to combine methodologies to provide a thorough, holistic understanding of how people respond to group singing experiences (in both real and virtual reality situations). However, in certain situations some of the techniques that have been used in isolation may prove impractical or intrusive. The following section provides an overview of some techniques which may be valuable in assessing parameters associated with health and well being in singing, considering their feasibility in certain experimental situations.

### 4.1. Indirect measures of health and well being

#### 4.1.1. Stress, arousal and enjoyment measures: UWIST

The University of Wales Institute of Science and Technology (UWIST) Mood Adjective Checklist (MACL) is used to determine acute subjective mood changes (Matthews, Jones, and Chamberlain 1990). The UWIST MACL is a 24-item checklist that gives acute measures of hedonic tone (valence), stress and (physical) arousal. In terms of singing research, the outcomes of stress and hedonic tone can be used as measures to understand the beneficial wellbeing effects of singing. The acute nature of the UWIST MACL means that researchers can understand changes in mood from subtle manipulations of experimental singing protocols and the questionnaires are easy and non-invasive to implement (taking approx. 30 seconds to complete).

#### 4.1.2. Neurological activity: EEG

While subjective assessments can be carried out during experimental singing protocols, there is the danger of participant fatigue and repetition effects that could be detrimental to the study outcomes. However, recent advances in mobile neuroimaging allow for real time recording of brain activity using relatively low-cost electroencephalography (EEG) headsets. The benefits of using these are that they are lightweight and send signals via bluetooth to an acquisition computer, ensuring participants are less restricted than they could be with more conventional, lab grade systems. The Emotiv EPOC+ headset has been validated for both indoor and outdoor use (Debener et al. 2012; Badcock et al. 2013) and shows real time brain activity that can indicate levels of relaxation (Kubitz and Pothakos 1997) and stress (Bonnet and Arand 2001) through increases in activity in specific signal bands (alpha and beta respectively).

#### 4.1.3. Heart rate and galvanic skin response: Shimmer

Shimmer GSR+ sensors provide an unobtrusive method to measure galvanic skin response (GSR) and blood volume pulse (BVP). GSR signals are measured by attaching two electrodes to the participant's palmar middle phalanges of the index and middle fingers on the non-dominant hand. The BVP signal is recorded using a photoplethysmograph that is attached to the same hand's ring finger. The GSR signal allows the extraction of the mean skin conductance level and mean number of skin conductance responses across a longer time period (Boucsein 2001). From the BVP signal, it is possible to extract the mean heart rate and the amount of sympathetic arousal-induced vasoconstriction. Additional measurements taken during a pre-singing resting period function as baseline measures. This allows calculation of individual change scores in physiological activation caused by singing as well as differences across conditions (e.g. across choir singing in the natural setting or the virtual setting). All extracted response descriptions indicate physiological activation or physiological deactivation that is typically associated with emotional responding (Scherer 2005), providing biophysical data to complement the UWIST data.

### 4.2. Measures of singing response

#### 4.2.1. Vocal fold behaviour: electrolaryngograph (Lx)

Electrolaryngographs allow measurement of the vocal fold activity of individuals as they sing, from which measures of pitch (tuning), closed quotient (connected to vocal efficiency) and amplitude can be made. Two small metal electrodes are placed externally on the neck at the height of the larynx and a low voltage, high frequency current is sent between them (for a literature review of research using electrolayngography in singing see (D'Amario and Daffern 2017)). This allows accurate non-invasive measures of individual voices to be taken in real-time whilst singing together. These data could provide insight into how vocal performance changes when people sing together and how these changes may map to other physiological and engagement data, especially considering the importance being placed on singing for voice therapy discussed in Section 1.2.

#### 4.2.2. Singing characteristics: DPA

Close proximity head-mounted microphones (DPA 4066) placed on the cheeks of the singers record their acoustic output at 16 bits and 44.1 kHz sampling frequency, to allow further scrutiny of the individuals' performances through spectral analysis where appropriate.

### 4.3. Measures of engagement

#### 4.3.1. Participant narratives: Sensemaker®

Quantitative measures of engagement are obtained after singing using Sensemaker®. Sensemaker® captures participants' individual stories as ‘micro-narratives’, which the participant subsequently interprets to reveal those elements of the experience which have most significance for them. A proven digital methodology for capturing and interpreting the complexity of social phenomena in ecologically valid ways (Snowden 2016; [Bibr CIT0050][Bibr CIT0050]), in this context the Sensemaker® method captures participants' experience of group singing, and translates these into more quantifiable data by a comparative analysis of ‘self-signified’ responses. These results can then be cross-referenced with other data to inform understanding of the participant experience and how these may be associated with health and well being.

#### 4.3.2. Engagement and practical considerations: impact questionnaires

Additional information about participant engagement can be obtained through questionnaires addressing specific research questions, such as whether they felt that they were really ‘there’, whether they felt like part of a shared experience, and whether they would engage with VR in the future based on their experience. Depending on the research question these can also be used to address methodological concerns such as whether the VR headset was comfortable, and whether the screen resolution was sufficient.

### 4.4. Technical considerations

It is important that whichever combination of measures taken throughout a performance (i.e. Shimmer, EEG, Lx, DPA) are synchronised for recording. All audio signals such as DPA and Lx can be automatically synchronised via an appropriate audio interface. However, the proprietary software required by e.g. Shimmers and Emotiv require a synchronisation signal to be recorded and used to align the data in post-processing.

## 5. Projects

The following section outlines the use of the above tools in different situations in three ongoing research projects. Table 1 summarises the measures obtained for each project.

**Table 1.** Data capture in the three research projects.

Measure:	UWIST	Sensemaker	Shimmer	EGG	Lx	DPA
Project 1	X		X	X	X	X
Project 2	X		X			X
Project 3		X				X

### 5.1. Ethical approval

Ethical approval was granted from the Physical Sciences Ethics Committee at the University of York for each project prior to commencement.

### 5.2. Project 1: singing quartet pilot

The purpose of this pilot was to test the feasibility of both the system design and implementation, and to test a protocol for measuring experience and health and well being responses of users of the system. The main research question of this project was: do singers respond in the same way when performing in the VR experience compared to a real performance situation? The pilot also considered how intrusive the different sensors described above might be for the participants, as well as how time-consuming and fatigue-inducing the questionnaire-based data collection might be. One semi-professional singing quartet (Soprano, Alto, Tenor, Bass) took part in this study.

#### 5.2.1. Experimental approach and preliminary findings

This project included the initial implementation of the VIIVA system reconstructing the experience of singing a performance in a vocal quartet in a church which is commonly used as a concert venue as described in Section 2. The user of the system becomes the fourth member of the singing quartet, hearing themselves in the church, and performing ‘live’ (but not interacting) with the other singers who can be seen and heard in the original space.

Two pieces were recorded: ‘Amazing Grace’ in unison, and Thomas Tallis's ‘If ye love me’ in four parts. Singers were asked to perform the songs as though it was a concert situation (although no audience was present at the time).

In order to minimise the effects of confounding variables, it was important that the same singers performed together in both the real performance and the VIIVA experience. For the VIIVA capture this required the original performance to be repeated four times, each with a different part missing and the camera and microphone recording in the place of the missing singer. Therefore, each singer took part in three recordings.

In addition to the recording of the VIIVA experience, the singers performed the singing tasks as a full quartet and data was collected from DPA, shimmer and Lx to measure their responses to performing in the real environment. UWIST questionnaires were filled in immediately before and after each performance, with baseline measures taken five minutes before and after the experiment. Baseline shimmer measurements were also taken prior to the recording taking place.

The singers each attended a separate session to test the VIIVA experience during which these measures were repeated, with the addition of EEG, to allow comparison of the singers' responses across the real and virtual environments. The data collected in this study are summarised in Table 1. Singers did not wear the EEG sensor during the live session, only in the VR condition, as the headset only became available after the live session had taken place. Singers reported that wearing the EEG headset didn't distract them and they forgot that they were wearing it.

It was desirable to apply a repeated measures design to this study in order to produce robust data, however, using a recorded VR experience this presented a particular challenge. In the natural environment, the singers performed each piece three times; whilst they did the same in the VR condition, only one VR recording was made with each part missing, so the repetitions in the VR condition were exact, whilst in the natural condition they varied. This is a feature of the VR experience which can be exploited for repeated-measures experimental design, however in this case when making comparisons between the two conditions, the discrepancy between the sets of repeated measures must be acknowledged. It was not practical to solve this problem by making three recordings per singer as it would have prohibitively lengthened the recording protocol, which already required half a day of recording time to produce a three-minute VR experience.

Whilst singers informally reported that they felt the same in the virtual environment as in the real venue, the results of the UWIST checklist show that they responded quite differently in the two conditions. In particular, on average, the singers' hedonic tone was generally lower but more stable in the VR condition, stress levels lowered noticeably over the live session whilst they remained high in the VR condition, and overall arousal increased over the course of the live session whilst remaining at a relatively low level throughout the VR session. These preliminary results suggest that perceptions of a performance experience may not be truly representative of the mood of the subject. This could be related to the theories of presence in VR mentioned in Section 2 and the expectations of the performers in the two conditions and is worthy of further investigation.

#### 5.2.2. Reflections on the protocol

This study focussed on within-subject comparisons, in part due to the limited number of singers, but also due to the limited resources available at the time. Only one heart rate and GSR sensor were available, so this data could only be captured for one singer at one time; this provided data for each singer across conditions, but prevented any cross-subject comparison. Singers reported that they didn't find the sensors intrusive, and that once they had been wearing them for a few minutes they weren't noticed. However, especially due to the use of individual microphones, the researchers were aware that the large number of cables (both attached to the singers and along the floor) reduced the ‘naturalness’ of the real environment as a traditional church performance venue for an acoustic choir performance.

Whilst singers reported that wearing the sensors was not disruptive to their experience, and, that the VR experience was ‘very real’, considering the different results of the singer's perceptions of their experience compared to their responses analysed via the UWIST questionnaires, further study is needed to methodologically assess the impact of the wearable technologies. The practical implementation of the EEG did not cause prohibitive problems in implementing the protocol, even though the singers wore a VR headset at the same time, which indicates that the Emotiv could be very insightful for singing research, especially as – given the necessary resource – multiple singers can wear them at once allowing a cross-subject as well as within-subject study design.

This project delivered a successful first VIIVA system alongside exploring methodologies for measuring response of the singers during performance. However, the expert vocal quartet does not necessarily lend itself to improving the accessibility of group singing across society, potentially being intimidating to a novice singer. Therefore future VIIVA experiences need to represent a community choir experience, especially if the health and well being of these groups is of interest.

### 5.3. Project 2: simulating inclusive natural group singing in virtual reality (SINGSVR)

The SINGSVR project extends the protocol developed for Project 1 to incorporate a ‘non-expert’ community choir rather than elite singers. The context of the VR experience also changed from taking part in a single ‘performance’ of a piece to being part of a rehearsal led by a director. To reflect the different purpose of the experience the venue changed accordingly to a typical community choir rehearsal venue. This project particularly sought to address the questions of whether the community singing experience could be reliably approximated by VR and whether the health and wellbeing benefits might be comparable in the two situations. This will shape future considerations in assessing whether a VIIVA experience could be of long term benefit to those otherwise unable to access a community choir.

It was not practical to implement the protocol from Project 1 – where an individual recording was made without each individual singer in the group – across a larger choir, as the number of VR recordings would need to be equal to the number of singers taking part. This was addressed in the SINGSVR project by engaging two choirs with a similar demographic, run by the same director and learning the same repertoire in the same way. One VR recording from within each choir was made and the participants experienced a VIIVA experience from the partner choir. This also ensured that they wouldn't see themselves in the recorded VR experience. The choir was arranged in a circle with singers of different ranges equally distributed throughout, to ensure that no one part was clustered around the recording equipment, affecting the VR experience. This solved the problem of repetition that was encountered in the pilot study: no recording was performed more than once by each singer, and the singers were introduced to new repertoire in both the VR and real environments to avoid learning effects influencing the results.

The choirs, consisting of two matched groups of amateur singers, undertook a longitudinal study across six weeks, in which they were taught six songs, three as real and three as VIIVA experiences (alternating conditions on a weekly basis). Singers were taught simple new material in each session, controlling for learning effects, and the same instructor, session format and choir arrangement were maintained for both groups. Sheet music cannot be used in the VIIVA condition as current VR headsets do not have sufficient resolution to reproduce it, so songs were always taught in such a way that they were learned and reproduced from memory.

The extended duration of the study allows singers to become accustomed to the VIIVA experience, which will provide information about the potential long-term health and wellbeing benefits of such a system while reducing the influence of disturbance due to the equipment.

#### 5.3.1. Reflections on the protocol

Using 12-person choirs in this study, with the same data acquisition across all singers (collected in parallel during the real condition and individually as they took part in VR) tested the potential to conduct research of this type with larger groups in a more ecological setting. The main limiting factor for such a protocol remains that of the time it takes to conduct the VR condition, as it has to be conducted individually for each participant. In this case, that resulted in the ‘real’ condition taking 120 minutes in total (three rehearsals of 20 minutes across two choirs), compared to the VR condition taking 24 hours in total (24 individual singers performing three sessions of 20 minutes each). Streamlining the data acquisition, in terms of making decisions as to the content and length of the rehearsals, the number of UWIST checklists and questionnaires, and which physiological data would be collected was essential, and resulted in a decision not to include Emotiv, or electrolaryngograph data in this study.

Attempting an ecological study of the rehearsal process of community choirs requires a substantial length of experiment in order for the experience to mimic that of a ‘real’ situation. Twenty minutes was chosen as a compromise between a normal rehearsal length (after discussion it was considered a suitable time of a rehearsal to include warm-up and work on a single, simple piece of music) and minimising the protocol length considering that the VR condition needed to be conducted one singer at a time.

At the centre of this project is a quasi-controlled experimental design investigating the health and well being benefits of group singing in order to further understanding of the impact of singing in VR physically separated from the other individuals. The following project has an emphasis instead on engaging wider communities with the VIIVA system and explore its potential to improve accessibility of different group singing scenarios.

### 5.4. Project 3: The Hills are Alive: combining the benefits of natural environments and group singing through immersive experiences

The Hills are Alive project, in partnership with the National Trust and Keswick Museum, focussed on increasing accessibility to choral experiences. Working with external partners is vital to maximise the engagement of the system with as diverse a target population as possible. A new VIIVA experience was created which provides participants' virtual access not just to a choir, but to geographically remote and inaccessible cultural locations.

This project archives a key moment in the formative history of the UK's National Trust: A choral piece, commissioned to commemorate the 1923 gift of land to the Nation in memory of those who lost their lives in the Great War 1914–18, performed on the top of Great Gable in the Lake District.

A 40 piece scratch choir was made up of community choirs from the local areas, who learnt the piece (and others) during rehearsal workshop days led by the conductor. The four-part ‘Fellowship of Hill and Wind and Sunshine’ (‘The Fellowship Song’), composed by Dave Camlin with words by Geoffrey Winthrope-Young, was recorded five times from the perspectives of within each voice part (soprano, alto, tenor, bass) and from an ‘audience position’ close to the conductor. In addition, a simple improvisation led by the conductor was also recorded from within the choir with voice parts mixed, in order to allow participants with no prior knowledge of the project to still join in with, and sing as part of, an experience. This resulted in five VIIVA experiences, comprising four voice parts and an audience position of ‘The Fellowship Song’, and one improvisation. Each experience began with a vocal warm-up led by the conductor which was recorded as part of each performance (so no edits were visible in the rendered experience).

It was not practical to capture the individual sensor measurements that were obtained in Projects 1 and 2 during the recording (which took place in the middle of a day's hiking) due to the bulk of the equipment, the number of participants, power and cabling requirements, and the time involved. All the participants were instead asked to complete a Sensemaker® story to capture their experience. 75% of stories submitted revealed a feeling of ‘a sense of sisterhood / brotherhood’ with ‘my people’ to be stronger than the ‘sense of place’, which is perhaps surprising given the significance of the landscape on which they were performing and the strenuous walk involved in getting there. Full analysis of the Sensemaker stories will be presented elsewhere as part of a larger study on group singing for well being.

Following the live recordings the five VIIVA experiences were installed at Keswick Museum for two full days to allow people to take part in the celebration through VR. The singers who took part in the recordings, singers who usually attend those choirs but couldn't take part in the live performance, as well as members of the general public, were invited to take part in the VIIVA version of the experience.

Of those taking part at the Museum, 13 participants knew ‘The Fellowship Song’ and performed their part within it whilst 9 more participants took part in the improvised piece and watched ‘The Fellowship Song’ from the audience position. After taking part in the VIIVA experience participants were asked about their experience of the VR and to complete a Sensemaker story. 86% of participants said that they would use VR again, 100% understood the instructions of the director within the experience, 27% felt inhibited at some point during the experience, 50% felt like they were on top of Great Gable (with 36% unsure), and 59% felt surrounded by the choir (with 41% unsure). The 21 stories submitted indicate more emphasis on ‘me’ than the stories collected in the original recording performances, suggesting not unexpectedly, that the VR experience is a more personal one. Comments of individuals indicate that the lack of haptic feedback (especially sensations of wind) contributed to reduced sense of being on the mountain summit, and that knowing the research assistant was in the room while they took part made some feel inhibited to sing. In terms of feeling surrounded by the choir, a couple of participants commented on feeling very ‘tall’: The set recording position of the camera prevents the perspective of the experience being altered to the individual taking part, although the recording deliberately included choir members standing on different levels of rocky ground to try to minimise the effect of this, it clearly wasn't always successful.

Whilst improving access was a key aim of this project and the installation at Keswick Museum enabled a number of people to experience unique group singing performances through VR, the challenges of presenting VR installations in a museum setting were also exposed. In particular as the VIIVA system includes interactive audio (whereby the user is required to make sound) an isolated space was needed to run the experience one participant at a time, reducing the number of people who could take part. The expense of the equipment and knowledge needed to run it also meant that the experience needed to be manned at all times, which is a costly resource. In order to engage the project with a wider audience a video showing the performance of ‘The Fellowship Song’ on a flat screen with stereo audio from the audience position was also installed as part of a larger exhibition of this and the National Trust's ‘Fellowship of Hill and Wind and Sunshine’ project which remained at the Museum for several weeks.

In an attempt to address the limitations of the expensive, cumbersome and complex system that was installed at the Museum, these VIIVA experiences were adapted for use with Samsung Gear headsets and phones. This version of the experience did not require the user to wear a microphone (which is needed for the real-time convolution of the acoustic), exploiting the acoustic of the original space (on a mountain summit) which had negligible impact on the sound of the experience as it is nearly anechoic. The chosen experience plays on a loop allowing users who have never used VR technology before to follow simple written instructions to put on the headset and headphones to take part. Three of these headsets were installed at the Lakes Alive Festival in Kendal, the Lake District, alongside the HTC Vive version that had been part of the Museum exhibit. The Samsung headsets ran the audience position of ‘The Fellowship Song’ whilst the Vive (supported by the research assistant) ran the improvisation. Over the festival weekend, 140 people tried the VIIVA experience. The self-assessment manikin was used to measure participant responses which 44 people completed (Bradley and Lang 1994). After using the experience, 12 felt excited / aroused; 17 felt somewhat excited; 11 felt calmer; 4 felt sleepier, with 100% of the responses on the positive end of the spectrum. Based on the Circumplex model of affect by Russell (1980) this suggests people felt happy, delighted, aroused and excited after the experience.

The positive overall feedback from the Museum and festival installations, which included a total of 162 people trying the VIIVA experience, indicates that it is a valuable tool to enable experience of group singing and natural environments, and, that further research should consider its potential to have a beneficial impact on the well being of individuals. To maximise its impact to enable access to group singing situations across a wider range of society, the challenges of resource (including time, equipment and expertise) need to be considered for future engagement with, and applications of, the VIIVA system.

## 6. Discussion

The positive reactions of users in all three projects indicate that the VIIVA system can provide a valuable tool for remote performance/ rehearsal of singing groups in both singing and ‘non-singing’ communities. The VIIVA has shown it can successfully provide remote access to group singing activities, which is a key goal of this research: Its installation at Keswick Museum and at the Lakes Alive Festival in Project 3 demonstrates that the current system is now portable and can be easily used and enjoyed by members of the public. The VIIVA system's current implementation across different platforms, including the HTC Vive and Samsung Gear provides an important step towards realizing a system that can be accessed using home technologies in the future. The feedback from users indicates that the isolation of the current system leads to a stronger sense of individual experience. As a known beneficial factor to group singing is connecting with others, development of the system in the future to allow real time interaction with others in the virtual environment is desirable to maximise its potential impact.

Project 2 shed light on the second main objective of this research to understand how users engage with singing in the VIIVA compared to a real situation. Apparent discrepancies between the four original singers' perceived levels of realism of the system compared to the real environment in Project 1, compared to their emotional responses as recorded by the UWIST checklist, indicates that understanding participants' experience of performance in the VIIVA system is an essential avenue of further enquiry. It is likely that the current restriction of the pre-recorded system, that the user cannot interact with the other singers in the environment, will have an impact on the sense of presence felt in the immersive experience, as described by Slater (2009).

It is especially important that future research considers the physical / psychological responses of users to the VIIVA system as well as their perceived responses, in order that the VR experiences can be tailored to their purpose. In particular, to understand the potential benefits of improving access to group singing through virtual environments, it is essential to understand how these responses relate to a real group singing experience: If the VR removes characteristics of a group singing experience that are vital to its beneficial impact then the technology or experiences will need to be adapted accordingly.

The uni-directional characteristic of the VIIVA system (in that the recorded singers cannot interact with the user in real time) has been shown to have potential to be exploited to control for social interaction in the study of the well being benefits of group singing as defined in objective three. Project 2 shows that a quasi-controlled experimental design is feasible, although highly time consuming to implement, and that a number of measurements can be taken in parallel during the singing experience. The combination of these data sets, may provide a holistic perspective of how people respond to group singing situations and how these might contribute to the perceived health and well being benefits of the activity. For a study such as this to be robust it is important the participants do not physically meet at any point when they attend their VIIVA session, in order not to re-introduce the social element that the VR is intended to control. This needs to be taken into account if multiple VIIVA systems (in acoustically isolated spaces) are being employed in parallel to improve efficiency of the study design by reducing the time taken to implement the VR condition.

Each project presents unique challenges and requires careful balancing of data acquisition, VR preparation and capture, ecology of design and resource management. In particular, utilising the VIIVA system as a tool for investigating the potential health and wellbeing benefits of group singing is costly to implement in terms of time: RIRs are required for any given position within an experience, in addition to the recording and rendering time. A key feature of the current system is that the interactive audio allows the user to perform as part of the recording. Unfortunately this prevents multiple singers from using the VIIVA system in the same space at the same time, as they would hear each other in the real space, detracting from the experience.

Whilst the current projects each focus on a different application of the system, including, development of the system itself, its use as a tool to control experimental environments, and how it can be practically implemented to improve access, together they are providing a promising foundation with which to develop the system and exploit its features to both maximise its impact across communities and investigate the health and well being benefits of group singing.

## 7. Conclusion

This paper introduces a new immersive experience, the VIIVA system, which enables the user to perform as part of a pre-recorded group singing activity in VR, hearing themselves within the recorded space. Whilst the audio is interactive the user cannot interact with the other singers in the environment. A set of tools and methods which can be employed to use the VIIVA system to study the health and well being benefits of group singing were presented. The practicalities of their implementation for different scenarios was discussed in connection with three ongoing research projects. Initial reactions to the system by users have been positive to date and indicate that the VIIVA in its current form could improve access to group singing for populations unable to attend live events. Preliminary implementations of the experimental designs suggest that the VIIVA is a promising tool for conducting research into health and well being benefits of group singing, especially by allowing control of factors relating to social interaction.
